# BARRIERS TO COMMUNITY FOOD RESOURCES AMONG OLDER ADULTS: PERCEPTIONS OF PROVIDERS AND OLDER ADULT STAKEHOLDERS

**DOI:** 10.1093/geroni/igae098.3530

**Published:** 2024-12-31

**Authors:** Sheryl Groden

**Affiliations:** University of MIchigan Flint, Flint, Michigan, United States

## Abstract

Finding proper nutrition for individuals living in or near a food desert is of particular concern for older adults, many who experience chronic health problems. This qualitative study explores barriers and challenges in food accessibility for older adults in Flint, Michigan. Qualitative data was collected at four focus group sessions in early 2022. Two focus groups included 15 older adults, 65 years of age or older. They shared their experiences and challenges regarding food accessibility. The other focus groups included six professionals employed at agencies connected to food distribution. Based on responses from both groups, we revealed challenges to food access/distribution among older adults. Data analysis highlights gaps in communication and knowledge of community food provision as barriers to older adults accessing food. Community providers noted while Flint doesn’t have adequate grocery stores, the community was not short of community-provided meals. Despite numerous food assistance programs in the community, older adults were unaware of all food distribution options. They were also unaware of digital apps to find food distribution or the best pricing for goods at grocery stores. As non-profit agencies and businesses rely more on digital communication, written communication is often forgotten, leaving out many older adults. Other identified communication barriers included literacy issues, language barriers (growing Arabic and Spanish-speaking populations), and lack of overall knowledge on where to go for community food. Implications for addressing gaps in communication will help to address food access and nutrition security for older adults in marginalized communities such as Flint, MI.

